# A Low-Cost Optoacoustic Sensor for Environmental Monitoring

**DOI:** 10.3390/s21041379

**Published:** 2021-02-16

**Authors:** Antonios Stylogiannis, Nikolaos Kousias, Anastasios Kontses, Leonidas Ntziachristos, Vasilis Ntziachristos

**Affiliations:** 1 Technical University of Munich, School of Medicine, Chair of Biological Imaging, 80333 Munich, Germany; stylogiannis@helmholtz-muenchen.de; 2 Helmholtz Zentrum München (GmbH), Institute of Biological and Medical Imaging, 85764 Neuherberg, Germany; 3 Mechanical Engineering Department, Aristotle University of Thessaloniki, P.O. Box 458, GR 54124 Thessaloniki, Greece; nkousias@auth.gr (N.K.); akontses@auth.gr (A.K.); leon@auth.gr (L.N.)

**Keywords:** photoacoustic, soot, black carbon, NO_2_, QTF, miniaturized, QEPAS, exhaust gas

## Abstract

Attention to Black Carbon (BC) has been rising due to its effects on human health as well its contribution to climate change. Measurements of BC are challenging, as currently used devices are either expensive or impractical for continuous monitoring. Here, we propose an optoacoustic sensor to address this problem. The sensor utilizes a novel ellipsoidal design for refocusing the optoacoustic signal with minimal acoustic energy losses. To reduce the cost of the system, without sacrificing accuracy, an overdriven laser diode and a Quartz Tuning Fork are used as the light source and the sound detector, respectively. The prototype was able to detect BC particles and to accurately monitor changes in concentration in real time and with very good agreement with a reference instrument. The response of the sensor was linearly dependent on the BC particles concentration with a normalized noise equivalent absorption coefficient (NNEA) for soot equal to 7.39 × 10^−9^ W cm^−1^ Hz^−1/2^. Finally, the prototype was able to perform NO_2_ measurements, demonstrating its ability to accurately monitor both particulate and gaseous pollutants. The proposed sensor has the potential to offer a significant economic impact for BC environmental measurements and source appointment technologies.

## 1. Introduction

Black Carbon (BC) is an important component of atmospheric particulate matter that has been recently attracting increased attention by the scientific community due to its effects on human health and the climate [1,2]. Regarding human health, BC concentration has been well-correlated with all-cause mortality as well as cardiovascular mortality and morbidity, due to its capacity to carry a wide variety of toxic chemicals [3,4]. Regarding its effects on the climate, BC is now considered to be the second most influential climate-altering agent next to CO_2_ [1,5] due to its high light absorption. Its effect is particularly important in remote snow or ice-covered areas (the Arctic, Himalayas, etc.) where it affects the albedo of the surface [6,7]. In contrast to CO_2_, BC is considered a short-lived climate-relevant species, rendering it an immediate target of regulation for fast climate recovery [2].

BC mostly comprises combustion-generated particles in the range of 20–200 nm formed by the pyrolysis of carbon-containing fuels [8,9]. Other species, such as metals from material wear or heavy organic species such as tar, may also contribute to what is measured as BC [10]. The difficulty in defining BC in terms of its physical or chemical properties has led to its definition—the most complete given by the International Maritime Organization [11]—being based on its strong light absorption properties across all visible wavelengths (e.g., in the order of 5 m^2^/g at 550 nm) and its carbonaceous nature. As the physical and chemical properties of BC constantly change during atmospheric aging, its light absorption properties also change significantly enough to affect precise measurements. The quantification of BC also depends on the operational principle used to measure its light absorption properties. As BC is mostly produced by combustion in vehicles, vessels, and stationary burners, its exhaust determination is necessary in order to control and therefore decrease the impact of these emission sources on the environment. Moreover, air quality monitoring of particles by means of sensor networks, which have recently attracted great attention [12,13], relies on sensors that can only detect large particles and exhibit lower accuracy compared to reference instruments [14].

Current particle measurement systems, such as condensation particle counters [15] and those based on electrical charging [16,17] and light scattering (nephelometers) [18], cannot distinguish between BC and other particle species. For distinguishing BC concentrations, optical absorption has been utilized in instruments such as opacimeters [19], aethalometers [19,20], as well as laser-induced incandescence (LII) systems [21]. Opacimeters measure the light extinction of a sample leading to measuring uncertainty due to scattering [22], while their sensitivity is low even for exhaust concentrations (≈300 μg/m^3^) [19]. Aethalometers are also affected by the light scattering of BC or other particles and gases, leading to measurement uncertainty [23]. Moreover, the absorption properties of BC particles change from deposition on the aethalometer filter, due to increased absorption of the scattered light by the soot cake [24]. While a miniaturized aethalometer was recently proposed [25], it was seen to underestimate BC concentrations with time, probably due to a loading artifact as the same filter is used for several days. For the same reason, it requires frequent maintenance interventions, as the filter needs to be frequently replaced. Finally, LII systems require high energy light sources to heat BC particles up to 4000 K [21], thus rendering the technology undesirable for a low-cost sensor.

Optoacoustic (also photoacoustic, OptA) sensors make use of the absorbed energy to create sound, thus achieving a signal that is proportional to light absorption [26]. Compared to other principles, the OptA principle has the greatest potential for accurate BC monitoring, with the operation principle falling in line with the BC definition based on its strong light absorption. However, significant size and cost reductions are still necessary before the technology can be implemented for large-scale environmental monitoring and source emission characterization. With current implementations of OptA devices for pollutants measurement, such as the AVL Micro Soot Sensor (MSS) [27], this is not possible, in particular because of the significant laser power required to achieve the necessary sensitivity. In addition, complex flow paths and secondary air flows are required to protect the sensitive components (optical and sound detectors) from contamination by the polluted sample flow. A recently proposed sensor [28] attempted to solve some of these issues by using a Quartz Tuning Fork (QTF) as a sound detector, which increases the sensitivity of the system, thus enabling a cheaper light source. However, this approach still suffers from contamination of the sensitive sound detector that is located in the path of the sample airflow and from the small size of the QTF, which significantly limits the size of the laser beam to approximately 300 μm [26]. Thus, possibilities for optimization of the laser beam size are severely limited in most cases.

The current study introduces a miniaturized and low-cost OptA sensor that provides an alternative to high-end equipment for the measurement of BC and potentially other gaseous pollutants. Due to the small size and reduced cost, more applications are available for the developed system such as air quality monitoring by means of a sensor network and an on-board exhaust monitoring system for specific sources. The sensor is based on a novel chamber design that refocuses the generated acoustic energy and by using cheap Laser Diodes (LD) that are overdriven for increased power output. A QTF is used as a sound detector due to its high quality (Q) factor. A prototype able to detect BC particles and nitrogen dioxide (NO_2_) was manufactured, which is able to perform real-time measurements with fast response to changes in BC concentration. This novel sensor represents a sensitive and more economic option among existing technologies, with the potential to significantly precipitate environmental pollutant measurements.

## 2. Materials and Methods

### 2.1. Sensor Design

OptA systems are widely used both in scientific efforts and in commercial applications. All these systems strive to offer the necessary sensitivity. Most OptA systems currently available make use of a cylindrical resonator in order to amplify the generated sound and achieve higher sensitivity [29,30,31]. By fine-tuning the geometry of the resonator, a significant increase of sensitivity can be achieved [32]. However, only a portion of the acoustic energy is captured by the acoustic detector due to its small area, and such systems exhibit significant dependence on the speed of sound and therefore operation and sample temperature. The AVL MSS that employs such a resonator requires a conditioning unit to control all sample parameters, including temperature. However, this leads to an increase in the size, complexity, and cost of the final system [27]. A different approach to increase the system’s sensitivity is to use mirrors in order to increase the light path distance, thus increasing the amount of absorbed light [33]. However, significant effort is required in order to protect the multiple mirrors and/or optical windows from contamination.

Quartz Tuning Forks (QTFs) have been used as sound detectors due to their high Q factor and small size in Quartz Enhanced Photoacoustic Spectroscopy (QEPAS) systems [26,34,35]. Systems that use a QTF as a sound detector commonly use either an on-beam or an off-beam configuration. In an on-beam system [26], the laser beam has to be focused between the QTF prongs, which have a distance of approximately 300 μm. Thus, there are requirements for accurate positioning of the QTF and the laser, and there is minimum potential for optimization of the laser beam size. Additionally, for a particle sensor, contamination of the QTF can result in a change in its resonant frequency with significant impact on the sensor’s operation. The off-beam approach overcomes these limitations by using a cylindrical resonator [36]; however, this causes other problems as mentioned above.

Therefore, existing approaches cannot offer the required sensitivity without significant drawbacks. A combination of solutions at different fronts is required to achieve a sensitivity similar to commercial instruments with significantly cheaper components.

**Figure 1:**
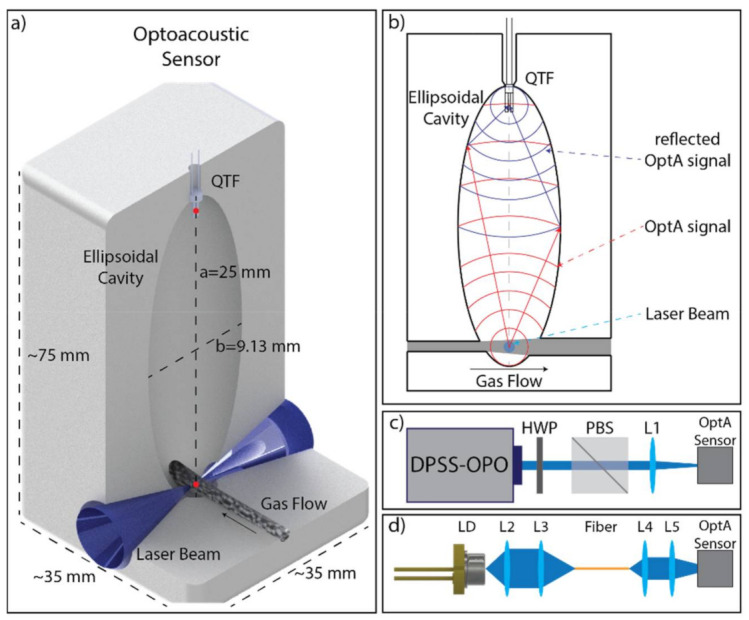
Prototype sensor and experimental setup used in the study. (**a**) Three-dimensional (3D) render of the acoustic chamber configuration across the light path cross-section and its dimension; (**b**) A 2D cross-section along the gas flow that shows the generation, reflection, and refocusing of the optoacoustic sound wave on the quartz tuning fork; (**c**–**d**) Configuration of the laser excitation systems used; (**c**) the DPSS-OPO laser setup; (**d**) the LD excitation system; DPSS-OPO: Diode Pumped Solid State-Optical Parametric Oscillator, HWP: Half-Wave Plate, PBS: Polarizing Beam Splitter; LD: Laser Diode; L1–L5: Lens 1–5.

In this work, a novel acoustic chamber is implemented that consists of a hollow ellipsoidal cavity [37]. Figure 1a shows a 3D render of the ellipsoidal cavity across the light path. The dimensions of the sensor are also presented and are appropriately scaled to the size of the QTF. The ellipsoid has two focal points (indicated with small red circles in Figure 1a); an acoustic signal generated at one of the two points is reflected off the walls of the ellipsoid and refocused at the other focal point, as demonstrated in Figure 1b. The ellipse has a unique property in that the distance between the first focal point, any point on the walls, and the second focal point is exactly the same. Therefore, ultrasound (US) waves converging at the second focal point have all traveled the exact same distance, independent of the speed of sound, frequency, and pressure, and they interfere constructively and in phase. Supplementary Figure S2 shows a visualization of the sound refocusing. Therefore, one can take advantage of this geometry to focus a laser beam at one focal point and concentrate the acoustic energy at the other focal point at a certain distance away. Thus, a strong signal is achieved, without the use of any resonators or amplifiers.

A constant gas flow is maintained through the acoustic chamber using a pump at the outlet of the sensor. The gas flow enters the chamber through a 2 mm diameter tube and exits through a 3 mm diameter tube that is positioned ≈6 mm away, at the other side of the ellipsoid, as shown in Figure 1a,b. In this way, minimal particle diffusion in the chamber is achieved. For 10 nm particles, the diffusion coefficient is equal to 5.4 × 10^−8^ m^2^/s, while the residence time in the chamber is ≈1 ms. These results indicate a mean particle displacement from thermal diffusion of 10 μm and estimated particle losses of less than 0.2% [38].

The gas flow crosses the laser beam at the first focal point. In this way, the detector, which is positioned at the other focal point away from both the light beam and the gas flow, is not being interfered with by the gas flow. The optical windows are positioned at the outer surface of the sensor away from the gas flow. The detector and the optical windows are expected to be protected from any contamination and subsequent degradation in sensitivity over time, due to the low particle diffusion achieved by the constant gas flow.

Moreover, the minimal particle diffusion ensures that there is no OptA signal generated out of the light–gas flow intersection point; i.e., no OptA signal is generated in the tubes that allow light and gas delivery in the chamber. Therefore, the proposed sensor does not act as a resonator as it does not amplify only specific frequencies, but it can be used to refocus the US wave of any frequency from the first focal point to the second. The attenuation of the US is expected to be ≈4 dB/m at 100 kHz and ≈0.8 dB/m at 32 kHz [39]. Therefore, the short US travel distance in the ellipsoid (≈50 mm) results in small sound attenuation of ≈0.2 dB at 100 kHz.

The ellipsoidal cavity of the prototype sensor demonstrated in the paper was designed and 3D-printed using an Inverse Stereolithography 3D printer (Form2, Formlabs, Somerville, MA, USA). Prototypes for testing were made of a plastic polymer.

A Quartz Tuning Fork (QTF) was selected as the sound detector due to its low cost, small size, and high sensitivity. By the novel design of the acoustic chamber, the usual limitation of the light beam diameter is overcome, and its value can be freely optimized. The two systems (light beam and sound detection) can be optimized independently, resulting in increased capabilities and improved final operation.

Laser Diodes (LD) were used as the light source, as their low cost and small form factor are particularly relevant for miniaturization of the sensor. The low energy output of the LD can be overcome since they can be driven with higher current to provide higher peak power than their continuous wave (CW) nominal values. This provides a significant advantage for OptA imaging and sensing, since the OptA signal requires fast transients [26,40,41,42]. The ability to use overdriven LD for OptA imaging has been demonstrated in [43]. In the current work, we used an updated version of the laser diode driver to overdrive the CW LD.

### 2.2. Experimental Setup

For the validation of the OptA sensor, we used two different light sources. First, a Diode Pumped Solid State (DPSS) laser equipped with an optical parametric oscillator (OPO) laser (Spitlight-DPSS 250 ZHGOPO, InnoLas, Krailing, Germany) providing 10 ns pulses at a repetition rate of 50 Hz was used due to its ability to fine-tune the excitation wavelength. The wavelength step was 5 nm, covering a range between 420 and 700 nm. To ensure constant pulse energy, ≈2 mJ per pulse, across all used wavelengths, a half-wave plate (HWP) was mounted on a motorized rotational stage (PRM1Z8, Thorlabs, Newton, NJ, USA), and a polarizing beam splitter (PBS) was used. The laser beam was focused in the OptA sensor, as described above, using a single plano-convex 75 mm lens (L1, LA1608-A, Thorlabs, Newton, NJ, USA). The setup is shown in Figure 1c. This laser source was used to assess the sensor performance with a well-characterized laser source.

Second, an overdriven CW LD at 462 nm (L462P1400MM, Thorlabs, Newton, NJ, USA) was used with the custom-made pulsed laser diode driver and controlled by an arbitrary waveform generator (33522B, Keysight, Newton, NJ, USA). The laser diode provided ≈7 ns pulses of 140 nJ at pulse repetition rates of ≈100 kHz, tuned to match the central frequency of the QTF. The use of nanosecond pulses ensures that the thermal and stress confinement limits are satisfied [44,45]. This is a low-cost light source that could be used in actual implementation of the sensor. To focus the output of the laser diode into a multimode fiber (M92L02, Thorlabs, Newton, NJ, USA), a two-lens system was used (L2 and L3, C340TMD-A and C560TME-A, Thorlabs, Newton, NJ, USA). The multimode fiber was used to reduce the strong electromagnetic interference generated by the pulsed laser diode driver, when triggered to create the current pulse, by physically creating distance between the driver and the OptA sensor. This also allows for the proper shielding and grounding of all electrical connections of the system, further reducing interference and noise. To focus the output of the fiber in the OptA sensor, a second two-lens system was used (L4 and L5, 15 mm D., 0.33 NA and 18 mm D. ×8 mm FL, Edmund Optics, Barrington, NJ, USA). The setup is shown in Figure 1d.

The OptA signal was captured by the QTF (100 kHz, Type TC-26, Conrad, Hirschau, Germany) and amplified by a custom-made amplifier circuit utilizing a dual operational amplifier (AD712D, Analog Devices, Norwood, MA, USA). We used a 100 kHz QTF because OptA signal generation is expected to be more efficient at higher frequencies [44,45]. The amplified signal was recorded using a high-speed digitizer (CSE1222, Dynamic Signals, Lockport, IL, USA), and the digitized signal was processed in Matlab (Matlab 2016b, Mathworks, Natick, MA, USA). The repetition rate of the pulse wave was tuned to be the same as the QTF resonant frequency, f0. The signal was recorded for 1 s at 500 kS/s. A Fast Fourier Transform (FFT) was performed, and the amplitude of the component at the frequency f0 was recorded. In this way, we perform digital lock-in detection with an effective detection bandwidth of Δf = 1 Hz.

### 2.3. Sample Preparation

**Figure 2:**
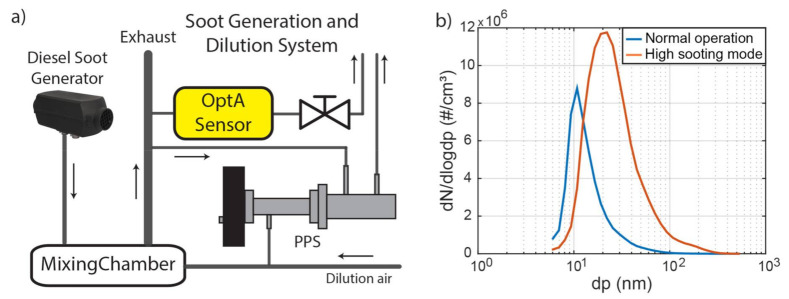
(**a**) The soot generation and dilution system. (**b**) The size distribution of particle number for two operation modes of the burner; dp: particle diameter, dN/dlogdp: number of particles in a specific range of particle diameters.

A planar 2D burner, operating on diesel, was used to generate BC particles for the experimental assessment of the sensor, as shown in Figure 2a. After the burner, a mixing chamber is used where the exhaust gas is diluted with clean and dry air. A low dilution ratio is used (1.2–2.5) to reduce the temperature and humidity of the exhaust while maintaining a high particle mass concentration. The sample is then carried to the sensor using conductive tubes. As a reference instrument, we used the Pegasor Particle Sensor (PPS), which determines the particle concentration by charging the suspended particles and measuring the current of the escaping gas flow [17].

At maximum power, the burner normally operates with lean combustion at a lambda value of 1.9. By reducing lambda to 1.6, an increase of both the particle number concentration and mean particle size can be achieved, increasing the overall concentration of BC produced. At such high BC output, lambda is only reduced for a limited amount of time (1–2 min) to prevent the buildup of BC deposits in the soot generation system. The operation with lambda equal to 1.9 is referred to as Normal Operation, and with lambda equal to 1.6 is referred as High Sooting Mode from now on.

The pollutants concentration at the exhaust of the burner was measured with high-end equipment to determine its composition: a HORIBA Portable Emission Measurement System (PEMS) was used to estimate the concentration of gases and the particle number (PN) concentration, an Engine Exhaust Particle Sizer (EEPS) was used to measure the particle number distribution, and an AVL MSS was used to measure the BC mass concentration. The composition of the exhaust is presented in Table 1. PN concentration was determined at 2 × 10^6^ #/cm^3^ and the BC concentration was determined at 100 μg/m^3^ at raw exhaust and Normal Operation. In High Sooting Mode, the BC concentration reached 1 mg/m^3^, while PN concentration levels were in the order of 1 × 10^7^ #/cm^3^. The particle size distribution as measured by EEPS is shown in Figure 2b, indicating that particles became larger and increased in concentration with decreasing lambda. The MMS and the PPS were used in parallel to characterize the exhaust gas of the burner and showed good linear correlation between them (R^2^ = 0.76, Supplementary Figure S1). In subsequent experiments, we used the PPS because of its portability and ability to provide a fast response time for sensor benchmark analysis.

**Figure 3:**
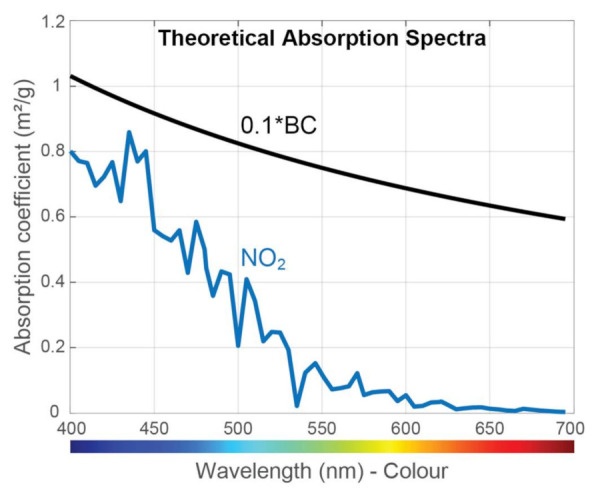
Theoretical light absorption intensity of Black Carbon (BC) together with the absorption potential of NO_2_ across the visible spectrum.

BC absorbs light across the visible region, and Figure 3 shows the theoretical absorption spectrum of BC based on the work of Bond and Bergstrom [24], who proposed an absorption cross-section of 7.5 m^2^/g at a wavelength of 550 nm, and an Angstrom coefficient equal to 1. In selecting the proper laser wavelength, other species that may also absorb in this range need to be considered. Of the relevant species listed in Figure 2, Figure 3 shows that NO_2_ absorption resides within the visible range and is stronger for blue light, according to the measurements of Schneider et al. [46]. No other relevant pollutants are expected to absorb significant light intensity in this range. Therefore, when selecting the proper light wavelength, we needed to choose between blue light, which is characterized by a strong BC signal but also NO_2_ interference, and red light, which is characterized by lower sensitivity but also zero interference from NO_2_. To demonstrate the ability of our sensor to detect BC, a LD at the blue wavelength was chosen because it provides higher power compared to a red LD [43].

## 3. Results

**Figure 4:**
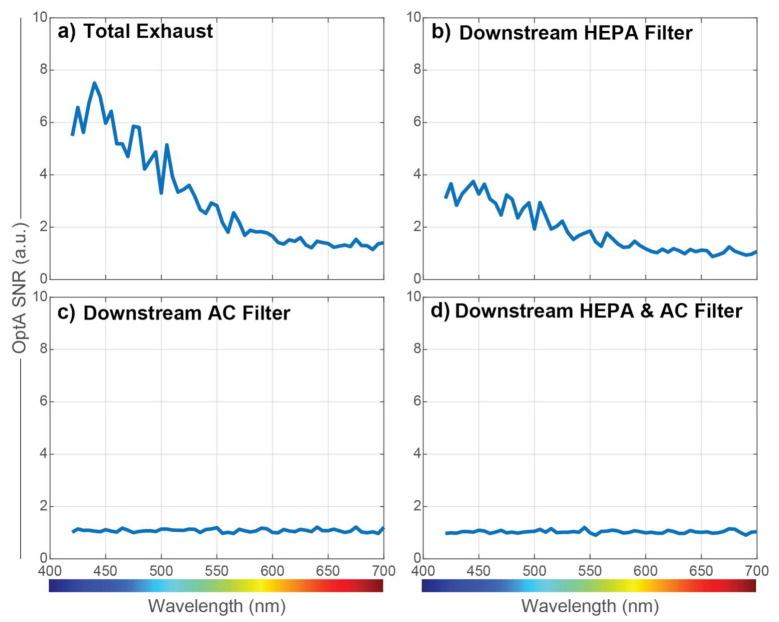
Sensor signal with different exhaust treatment. (**a**) Total exhaust, (**b**) Exhaust filtered by a High-Efficiency Particulate Air (HEPA) filter, (**c**) Exhaust filtered by an Activated Carbon filter, (**d**) Exhaust filtered by a HEPA filter and an Activated Carbon Filter in series.

The first setup with the DPSS-OPO laser was used in order to evaluate the OptA spectrum of the exhaust gas. As mentioned, light absorption is expected to arise from both BC particles and NO_2_. First, we exposed the sensor to unfiltered exhaust to obtain information of total light absorption produced by the exhaust gas, as presented in Figure 4a. Then, in a second experiment (Figure 4b), we exposed the sensor to exhaust which has been filtered using a High-Efficiency Particulate Air (HEPA) filter with a particle removal efficiency of >99.9%. In a third experiment (Figure 4c), an Activated Carbon (AC) filter was used to filter out the gaseous components, including NO_2_. Finally, we exposed the sensor to exhaust which has been filtered using both a HEPA filter and an AC filter in series (Figure 4d), aiming to remove all absorbing species. The burner was set to Normal Operation in all cases, and the SNR of the OptA signal was calculated [47,48].

Figure 4a shows the absorption spectrum of both BC and NO_2_, while Figure 4b shows the spectrum of only NO_2_, as most particles were captured by the HEPA filter. The absorption in Figure 4a, where both BC and NO_2_ are present, was stronger than in Figure 4b, where only NO_2_ is present, for all wavelengths. This demonstrates that BC absorbs in all visible wavelengths, which is in agreement with the theoretical values in Figure 3. No spectra were recorded when the AC filter was used, as it also removes BC at low concentrations. Additionally, the used DPSS laser was emitting pulses at 50 Hz without the ability to fine-tune its repetition rate, resulting in low sensor sensitivity because no harmonic of its repetition rate coincides with the central frequency of the QTF, as shown below. Finally, with both filters in place and BC, NO_2_, and other light absorbing species removed from the flow, the OptA signal was reduced to background levels for all frequencies.

The same experiment for the absorption spectra of the exhaust was performed with a second prototype and gave similar results (data not shown). Both prototypes were assembled by hand without using any micro positioning stages or other mechanism for exact positioning. This indicates that due to the ellipsoidal chamber, the sensor is not sensitive to the exact location of the QTF. This can be explained by the fact that refocusing sound at low frequencies, 10–200 kHz, results in a relatively large acoustic focal area, in the order of mm. The size of the OptA focal spot for that frequency is independent of the size of the ellipsoid (Supplementary Figures S2 and S3). As long as the QTF is located in this relatively large focal area, the response of the sensor is not affected.

**Figure 5:**
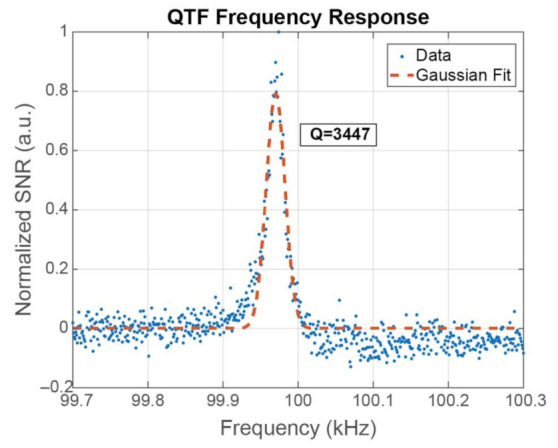
Frequency response of the Quartz Tuning Fork and the sensor’s Q factor.

We next used the second light source, the overdriven LD, to illustrate that such a low-cost light source can be used to detect BC particles at low concentration. First, the frequency of the light beam was varied by 1 Hz between 99.7 and 100.3 kHz in order to identify the exact resonant frequency of the QTF and evaluate the sensor’s Q factor. This experiment was performed twice: first with clean and dry air and then with the sample from the burner. The signal-to-noise ratio (SNR) was calculated as the amplitude of the particles signal over the standard deviation of the noise at each frequency [47]. The burner was operated in Normal Operation. Figure 5 present the normalized (SNR) of the sensor’s response that was almost zero outside of a small interval between approximately 99.9 and 100 kHz. The maximum response was at a laser pulse repetition rate of 99.974 Hz. The location of maximum response was a few Hz lower than 100 kHz, since the QTF was operating in air and not in vacuum. The Q factor was calculated by a Gaussian fit of the data to be equal to 3447, which is approximately 2–3 times lower than its normal value in atmospheric pressures, which is usually >10,000 [26,47]. The main reason for this is that the detection bandwidth, Δf, of the QTF is relatively large at ≈29 Hz because the proposed sensor does not act as a resonator.

**Figure 6:**
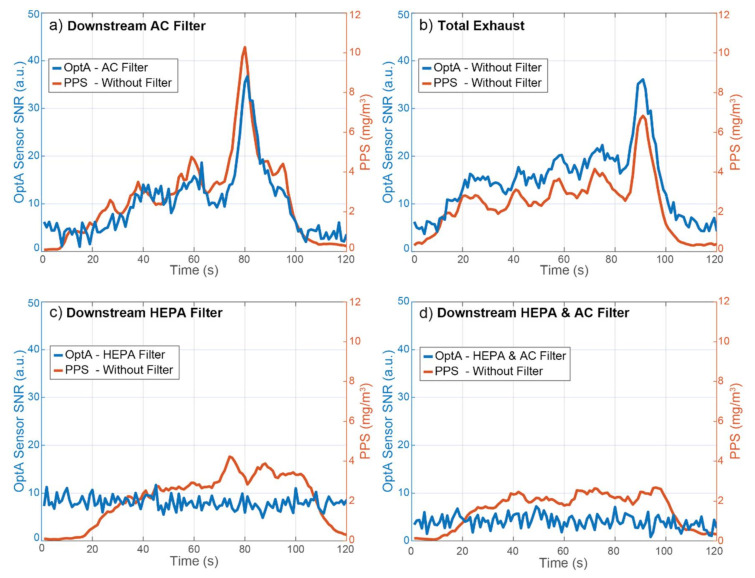
Sensor signal-to-noise ratio (SNR) in High Sooting Mode and comparison with a Pegasor Particle Sensor (PPS), used as a reference instrument. The PPS always received an unfiltered sample. (**a**) Optoacoustic (OptA) Sensor’s sample filtered by an Activated Carbon Filter, (**b**) total exhaust, (**c**) OptA sensor’s sample filtered by a HEPA filter, (**d**) OptA sensor’s sample filtered by a HEPA filter and an Activated Carbon Filter.

Next, the performance of the sensor was evaluated by operating the burner at High Sooting Mode for 1 min at a time. During that operation, the NO_2_ concentration increased slightly, while the particle concentration increased by an order of magnitude (Figure 2 and Table 1). The BC concentration was also unstable, leading to significant second-by-second changes. The response of the OptA sensor under such conditions was compared to that of the PPS reference instrument, and the OptA sensor’s SNR along with the PPS response are presented in Figure 6. That experiment was initially performed with the activated carbon filter to eliminate the NO_2_ signal (Figure 6a). The drastic changes in concentration were captured by both instruments equally well, pointing to the excellent performance of the prototype due to its ability to detect changes in concentration in real time and with great accuracy. The same experiment was subsequently performed a second time without using any filters (Figure 6b). A similarly positive performance was observed for the OptA prototype. The signal of the prototype was higher relative to PPS, which is something that can be attributed to the extra response to NO_2_, which was not completely eliminated.

Figure 6c demonstrates the OptA sensor readings after use of the HEPA filter during a third experiment, which eliminates particles, and the PPS response to the unfiltered sample. An increase in particle concentration similar to Figure 6a,b was observed based on the PPS measurements. However, the OptA sensor’s signal remained constant, which can be attributed to NO_2_, which is stable in High Sooting Mode. Figure 6c confirms the results, indicating that the signal of the OptA sensor in Figure 6a,b was due to particles, since no signal increase was observed when the particles were filtered. Finally, Figure 6d shows the response when the OptA sensor was used downstream of both a HEPA and an Activated Carbon Filter during a fourth experiment. A similar curve compared to the previous experiments was seen for the PPS. The OptA sensor had no signal during this experiment.

**Figure 7:**
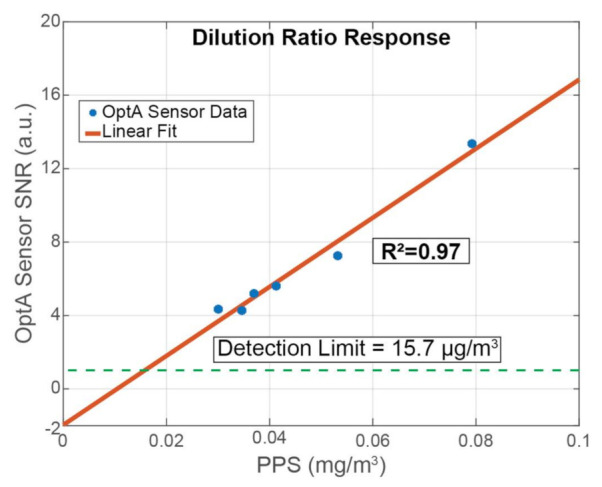
Response of the sensor under different dilution ratios and comparison with the PPS.

Finally, the dilution ratio was varied, and 30 consecutive 1-s measurements of the OptA sensor were averaged and compared to the average particle concentration measured by the PPS at the same time to determine the BC detection limit of our sensor. For that experiment, the burner was set to Normal Operation, and the dilution ratio changed from 1.3 to 2.4. The correlation between the prototype’s SNR and the PPS was calculated and plotted along with the data in Figure 7.

The BC concentration varied between 30 and 80 μg/m^3^ depending on the dilution. The present soot generation and dilution system could not achieve a higher dilution or lower concentrations. Higher concentrations were also not possible, as operating the soot generator at High Sooting Mode causes significant and fast changes in the BC concentration and the particle size distribution. No filter was used for that experiment because the AC filter also removes BC at low concentrations, as shown above; therefore, there was an added response due to NO_2_. According to the spectra measurements performed above, in Normal Operation Mode, the contribution of BC in total, from BC and NO_2_, was approximately 44%. Additionally, according to the exhaust gas characterization and the correlation between the PPS and the MSS (Supplementary Figure S1), we observed the PPS detecting BC concentrations approximately 4-fold higher compared to the MSS. This is due to the PPS being calibrated for different particle size distribution. We took into account both these factors to determine the BC concentration limit.

A good linear correlation was observed with an R^2^ value of 0.97 that can be even higher if a wider concentration range is used. The linear correlation yielded a non-zero offset value (≈−2 (a.u)) that increases the detection limit, which can be a result of additional noise or interference from other sources that reduce the sensor’s SNR. Thus, the normalized noise equivalent absorption coefficient (NNEA) [47] for BC is calculated to be 7.39 × 10^−9^ W cm^−1^ Hz^−1/2^ and the Noise Equivalent Concentration (NEC) is calculated to be 15.7μg/m^3^.

## 4. Discussion

Black Carbon is a pollutant that degrades air quality, is adverse for human health, and significantly contributes to global warming. Despite its tremendous importance, there are currently no low-cost systems for its detection in the field or at an individual source level. In this paper, we presented a novel OptA sensor that can achieve the detection of BC particles at low concentrations and in real time using low-cost components. Such a system can provide a cheap alternative to current instrumentation for a variety of implementations.

Several new features were combined to reduce the size and cost of current OptA technology for BC sensing. First, an ellipsoidal cavity was used to accommodate all the sensor components, which allows for efficient sound detection by concentrating the generated acoustic energy. Second, overdriven Laser Diodes were used to achieve an efficient compromise between cost and high light peak power. Finally, a Quartz Tuning Fork was selected as the sound detector due to its high Q factor. The manufactured prototype was able to detect BC and NO_2_ for the selected laser wavelength and demonstrated a very fast response to changes in concentration. Additionally, two prototypes were assembled by hand and exhibited the same response, indicating that an exact positioning of the sound detector is not required, thus reducing manufacturing effort and minimizing sensitivity to mechanical vibrations.

An additional advantage of the ellipsoidal cavity is that it concentrates acoustic energy rather than amplifying a sound wave. While it increases sensitivity, the use of acoustic amplifiers such as resonators in existing applications results in great temperature dependence as the speed of sound varies with temperature. As a result, a conditioning unit is necessary, which increases size, cost, and complexity. By concentrating the acoustic energy, these problems can be overcome because the speed of sound does not influence the focusing ability of the proposed sensor. Moreover, the ellipsoidal design is expected to protect all sensitive components. First, the QTF is located several millimeters away from the gas flow, thus being protected from particle contamination that would affect its long-term operation. Second, the optical path is perpendicular to the gas flow, also limiting BC deposition on any optical windows and the need for regular cleaning. Finally, the ability to couple the light beam in an optical fiber allows the LD to be further away from the measuring unit. Thus, the ellipsoid can be positioned in a high temperature area without affecting the LD’s functionality.

The OptA sensor achieved a detection limit for BC of 15.7 μg/m^3^ and an NNEA of 7.39 × 10^−9^ W cm^−1^ Hz^−1/2^, which is comparable to other QEPAS systems that usually achieve an NNEA in the order of 10^−8^ to 10^−10^ W cm^−1^ Hz^−1/2^ [47]. However, the BC detection limit could not be properly characterized due to several factors. The exhaust gas also contained NO_2,_ the contribution of which in the measured SNR had to be mathematically eliminated. In addition, the PPS reference system works on a different principle based on diffusion charging compared to optoacoustic, and its measurements had to be readjusted to fit the MSS measurements. Moreover, the existing dilution system only offered a limited dilution range, which reduced the accuracy of the linear fit. Despite these limitations, the detection limit measured is expected to be in the correct order of magnitude and is considered to be sufficient for a newly developed prototype.

The Q-factor of the sensor was two to three times lower than expected because it refocuses the US rather than acting as a resonator. However, even compared to on-beam QEPAS systems [34], the Q-factor is still lower and might be due to the nature of the 100 kHz QTFs, which have not been extensively tested in QEPAS so far. Future studies will aim at further improving the Q factor and therefore the sensor’s sensitivity. The same sensor, with minimal changes, can also be used with a 32 kHz QTF. Additionally, different ellipsoid sizes can be tested and evaluated to reduce the sensor’s detection limit. The next steps also include the use of a Lock-in Amplifier, calculation of the Allan deviation to determine the optimal averaging time, and the use of a sensor made from metal to reduce losses when the sound is reflected off the walls of the ellipsoid. Additionally, a multi-wavelength system is also possible, since multiple light beams can be coupled in the same optical fiber. Such a multi-wavelength sensor could be used to detect multiple species simultaneously.

In summary, the developed sensor has the potential to fulfill the function of a sensitive, low-cost BC sensor. Specifically, the cost of the QTF is negligible, while the LD has a maximum cost of a few hundred euros. Conventional light sources typically cost in the order of tenths of thousands of euros, highlighting the tremendous potential for cost reduction of the technology. The final cost for mass production is expected to be in the order of hundreds of euros. The sensitivity of the prototype is calculated to be 15.7 μg/m^3^. By further improving the system, we aim for a detection limit of 1 μg/m^3^ or even lower, which would render the technology adequate for atmospheric measurements.

In addition to improved sensitivity, by proper selection of the wavelength to exclude the NO_2_ absorption range, one can specifically isolate BC, which would be needed for atmospheric sensor networks. Additionally, the prototype is already capable of evaluating the proper function of exhaust post-treatment systems, which require a detection limit of approximately 100 μg/m^3^. For such direct vehicle exhaust applications, selecting blue light would make the sensor responsive to both BC and NO_2_. This would be advantageous, because a single sensor could be used as an on-board monitoring device and detect malfunctions of both the particle filter (that would increase BC) and the NO_2_ post-treatment devices. Other wavelengths could be used for other pollutants, for example, CO_2_ at 1370 nm. It is important to be able to monitor the CO_2_ emissions of vehicles during their operation, but there is currently a lack of low-cost CO_2_ sensors for measuring exhaust pollutant concentrations. Therefore, the proposed sensor offers promising potential for an array of future environmental monitoring applications.

## Figures and Tables

**Table 1:** Exhaust gas content of individual pollutants in Normal Operation and High Sooting Mode.

Pollutant	Normal Operation λ = 1.9	High Sooting Mode λ = 1.6
CO_2_ (%)	7.2	9.8
CO (ppm)	84	475
NO_2_ (ppm)	7.6	9
NO (ppm)	44	57
HC (ppm C)	0	2.2
PN [CPC] (#/cm^3^)	2.1 × 10^6^	1.3 × 10^7^
BC [MSS] (mg/m^3^)	0.1	1.7

## Data Availability

The data presented in this study are available in this article and the supplementary information.

## Supplementary Materials

The following are available online at https://www.mdpi.com/1424-8220/21/4/1379/s1, Figure S1: Correlation of the Pegasor Particle Sensor (PPS) with the AVL Micro Soot Sensor (MSS) for the measurement of particles that are created from the BC generation system in Normal Operation, Figure S2: Results from the Computational Fluid Dynamics (CFD) simulation that models the propagation of ultrasound waves and the formation of a high-pressure volume at the second focal spot of the ellipse, Figure S3: Size of the acoustic focal area as a function of the ellipse major axis length.

## Patents

An application for a patent license (GR20200100657) has been submitted for the optoacoustic sensor presented in this paper.
