# Supplementary material for: A Low-Cost Optoacoustic Sensor for Environmental Monitoring
Source: Sensors (Basel). 2021 Feb 16;21(4):1379. doi: 10.3390/s21041379 (PMC7920291; doi:10.3390/s21041379)
Supplement: Supplementary file 1 [file sensors-21-01379-s001.pdf]

Supplementary Material

PPS – MSS correlation

An experiment was run, where both the AVL Micro Soot Sensor (MSS) and the Pegasor Particle Sensor (PPS) were used simultaneously to measure the particle concentration of the BC generation system's exhaust. During the experiment, the BC generation system was set in Normal Operation and the dilution was varied. At supplementary Figure 1 we observe that there is a good linear correlation between the two instruments, but the R^2 is relatively low. That can be explained by the fact that the two instruments have different operating principles. Additionally, the concentration measured by the PPS is approximately 4 times higher than the one measured by MSS. That can be attributed to the fact that the PPS is calibrated for particles of ~ 50 nm diameter, and it produces an error for particles of different diameter. Specifically, it overestimates the concentration of smaller particles and it underestimates the concentration of larger particles. In our sample, most particles were below 20 nm in size (smaller than the PPS is calibrated) and thus the PPS overestimates their concentration.

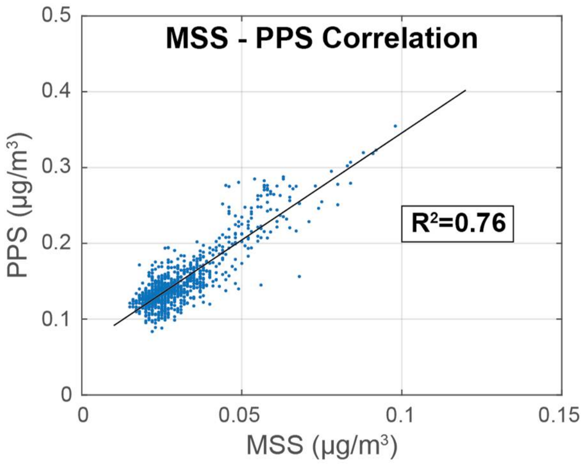

Supplementary Figure 1: Correlation of the Pegasor Particle Sensor (PPS) with the AVL Micro Soot Sensor (MSS) for the measurement of particles that are created from the BC generation system in Normal Operation

Sound Refocusing

CFD simulations were run to evaluate the refocusing of ultrasound (US) waves by the ellipsoid chamber. The geometry of the chamber in the simulation is identical to the geometry of the prototype. To reassure the accuracy of the simulation, a mesh independence analysis was performed, in which the parameters of the boundary layer were also considered. The optoacoustic generation was simulated as a pulsed energy input with a repetition rate of 100 kHz, in a 2 mm sphere that is located at the first focal spot of the ellipse (not visible in Supplementary Figure 2). In order to better visualize the fluctuations of pressure due to sound propagation, the simulation was run without sample flow in the chamber.

In Supplementary Figure 2 it is apparent that the intensity of the ultrasound has weakened at the center of the ellipsoid chamber, but its intensity increases again at the location of the second focal spot. The existence of such a high-pressure volume proves that the ellipsoid refocuses the acoustic energy from the first focal spot to the second.

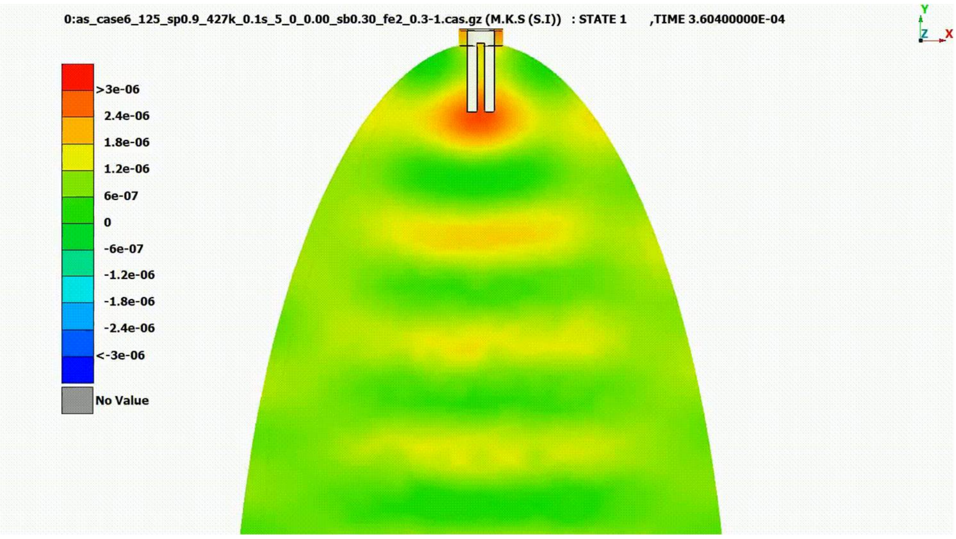

Supplementary Figure 2: Results from the CFD simulation that models the propagation of ultrasound waves and the formation of a high-pressure volume at the second focal spot of the ellipse

Size of the acoustic focal area

The same CFD simulation was run for different ellipsoid geometries with variable length of the ellipse major axis. The results show that the same profile is observed in all cases, with a focal acoustic area of approximately 2.5 mm in diameter. Smaller (~ 10 mm) or significantly larger (~ 200 mm) ellipsoid chambers are not considered relevant for the intended application.

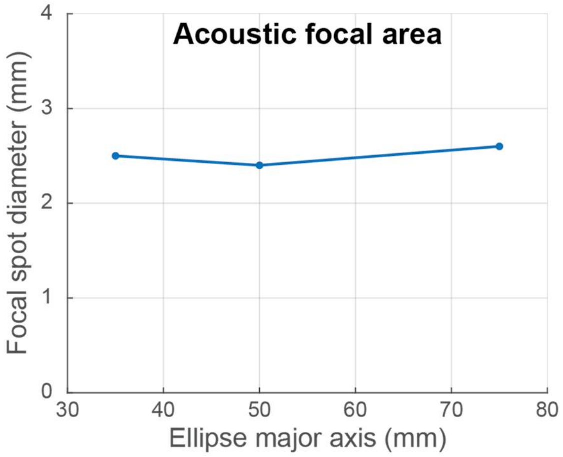

Supplementary Figure 3: Size of the acoustic focal area as a function of the ellipse major axis length
